# Aggressive Multiple Myeloma with Unusual Morphology

**DOI:** 10.4274/tjh.2013.0104

**Published:** 2014-12-05

**Authors:** Mehmet Sönmez, Hasan Mücahit Özbaş, Nilay Ermantaş, Ümit Çobanoğlu

**Affiliations:** 1 Karadeniz Technical University Faculty of Medicine, Department of Hematology, Trabzon, Turkey; 2 Karadeniz Technical University Faculty of Medicine, Department of Pathology, Trabzon, Turkey

**Keywords:** Multiple myeloma, Unusual morphology

## MORPHOLOGY IN HEMATOLOGY

A 66-year-old male patient was admitted to the hospital with backache, fatigue, and paraesthesia and spasm in both legs. He had lower extremity numbness and bladder and bowel incontinence. Physical examination revealed the absence of bilateral lower extremity reflexes and lower extremity weakness. Magnetic resonance imaging showed a large mass extending from T8 to T9, fracture of T9, and compression of the spinal cord. Informed consent was obtained.

**Figure 1:**
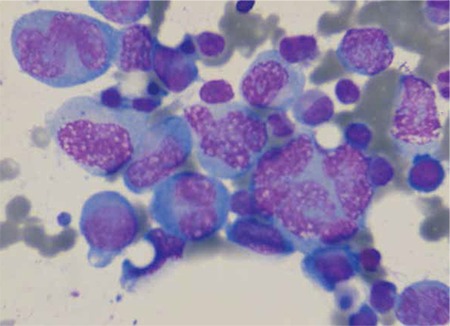
Atypical and multinucleated plasma cell infiltration in the bone marrow aspirate (Wright’s stain, 100x).

**Figure 2:**
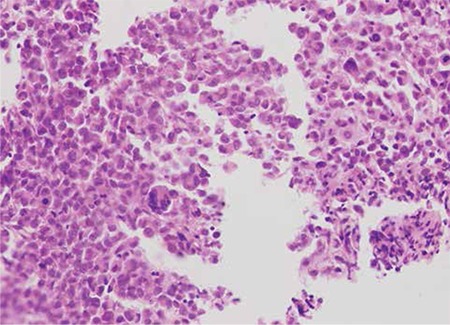
Atypical and multinucleated plasma cell infiltration in the bone marrow biopsy (H&E, 20x).

Laboratory results at initial evaluation revealed the following: haemoglobin: 118 g/L, haematocrit: 33.5%, white blood cell count: 6.7x109/L, platelets: 192x109/L, blood urea nitrogen: 7.5 mmol/L, creatinine: 113.1 µmol/L, calcium: 1.9 mmol/L, total protein: 63 g/L; albumin: 31 g/L; and erythrocyte sedimentation rate: 59 mm/h. Protein studies by nephelometry revealed IgA of 4.25 g/L (reference range: 0.7-4 g/L) and lambda light chain of 3.98 g/L (reference range: 0.9-2.1 g/L). A small monoclonal spike was present upon protein electrophoresis. Urine immunoelectrophoresis documented no monoclonal light chain. Bone marrow aspirate and biopsy were performed and the patient underwent surgical decompression and stabilisation of the thoracic spine. The bone marrow aspirate and biopsy morphology showed infiltration with atypical, multilobated, cleaved, and monocytoid nuclei plasma cells (Figures 1 and 2). The biopsy material stained positive with lambda light chain and CD138.
